# Association Between Therapeutic Alliance and Outcomes Following Telephone-Delivered Exercise by a Physical Therapist for People With Knee Osteoarthritis: Secondary Analyses From a Randomized Controlled Trial

**DOI:** 10.2196/23386

**Published:** 2021-01-18

**Authors:** Belinda Joan Lawford, Kim L Bennell, Penny K Campbell, Jessica Kasza, Rana S Hinman

**Affiliations:** 1 Department of Physiotherapy Centre for Health, Exercise and Sports Medicine The University of Melbourne Melbourne Australia; 2 Department of Epidemiology and Preventive Medicine Monash University Melbourne Australia

**Keywords:** osteoarthritis, physiotherapy, physical therapy, tele-rehabilitation, telephone, therapeutic alliance, exercise, knee, pain

## Abstract

**Background:**

The therapeutic alliance between patients and physical therapists has been shown to influence clinical outcomes in patients with chronic low back pain when consulting in-person. However, no studies have examined whether the therapeutic alliance developed between patients with knee osteoarthritis and physical therapists during telephonic consultations influences clinical outcomes.

**Objective:**

This study aims to investigate whether the therapeutic alliance between patients with knee osteoarthritis and physical therapists measured after the second consultation is associated with outcomes following telephone-delivered exercise and advice.

**Methods:**

Secondary analysis of 87 patients in the intervention arm of a randomized controlled trial allocated to receive 5 to 10 telephone consultations with one of 8 physical therapists over a period of 6 months, involving education and prescription of a strengthening and physical activity program. Separate regression models investigated the association between patient and therapist ratings of therapeutic alliance (measured after the second consultation using the Working Alliance Inventory Short Form) and outcomes (pain, function, self-efficacy, quality of life, global change, adherence to prescribed exercise, physical activity) at 6 and 12 months, with relevant covariates included.

**Results:**

There was some evidence of a weak association between patient ratings of the alliance and some outcomes at 6 months (improvements in average knee pain: regression coefficient −0.10, 95% CI −0.16 to −0.03; self-efficacy: 0.16, 0.04-0.28; global improvement in function: odds ratio 1.26, 95% CI 1.04-1.39, and overall improvement: odds ratio 1.26, 95% CI 1.06-1.51; but also with worsening in fear of movement: regression coefficient −0.13, 95% CI −0.23 to −0.04). In addition, there was some evidence of a weak association between patient ratings of the alliance and some outcomes at 12 months (improvements in self-efficacy: regression coefficient 0.15, 95% CI 0.03-0.27; global improvement in both function, odds ratio 1.19, 95% CI 0.03-1.37; and pain, odds ratio 1.14, 95% CI 1.01-1.30; and overall improvement: odds ratio 1.21, 95% CI 1.02-1.42). The data suggest that associations between therapist ratings of therapeutic alliance and outcomes were not strong, except for improved quality of life at 12 months (regression coefficient 0.01, 95% CI 0.0003-0.01).

**Conclusions:**

Higher patient ratings, but not higher therapist ratings, of the therapeutic alliance were weakly associated with improvements in some clinical outcomes and with worsening in one outcome. Although the findings suggest that patients who perceive a stronger alliance with their therapist may achieve better clinical outcomes, the observed relationships were generally weak and unlikely to be clinically significant. The limitations include the fact that measures of therapeutic alliance have not been validated for use in musculoskeletal physical therapy settings. There was a risk of type 1 error; however, findings were interpreted on the basis of clinical significance rather than statistical significance alone.

**Trial Registration:**

Australian New Zealand Clinical Trials Registry ACTRN12616000054415; https://www.anzctr.org.au/Trial/Registration/TrialReview.aspx?id=369204

## Introduction

### Background

Knee osteoarthritis (OA) is highly prevalent and leading cause of functional limitation in older adults [1,2]. Given that there is no cure for OA, long-term self-management of the condition aims to reduce joint pain and improve physical function and quality of life. All current clinical guidelines recommend education, exercise, and if appropriate, weight loss [3-6]. Physical therapists are one of the most common providers of exercise management for people with OA [7] and traditionally, consultations occur in-person at a physical therapy clinic. However, there is a growing body of literature to support the safety and effectiveness of tele-rehabilitation, where physical therapists and patients consult remotely using telecommunication technologies, such as video conferencing or telephone [8-10]. Accordingly, tele-rehabilitation, as the mode of delivery of physical therapy services, is increasingly being advocated and implemented in Australia [11], the United Kingdom [12], and the United States [13,14].

An important aspect of health care is the strength of the relationship developed between the patient and the health professional. This relationship, known as the therapeutic alliance, is conceptualized as a sense of collaboration, warmth, and support between a patient and clinician [15], and it focuses on 3 elements of the relationship: (1) agreement on goals; (2) agreement on tasks; and (3) personal bond. Extensive research in psychotherapy settings (eg, patients recovering from schizophrenia, poor mental health, drug use) has demonstrated that a strong therapeutic alliance between patients and their therapists can positively influence satisfaction with care, quality of life, psychological well-being, symptom improvement, and treatment adherence [16-19]. There is emerging evidence that therapeutic alliance is also important in musculoskeletal rehabilitation. Two recent systematic reviews found evidence that a therapeutic alliance in people with chronic musculoskeletal pain (eg, chronic low back pain) was associated with clinical outcomes from treatment, including improvement in pain and function [20,21]. In contrast, another systematic review reported that the small number of studies available, failed to provide evidence of a strong relationship between therapeutic alliance and improvement in pain [22]. None of the studies cited in any of these three reviews evaluated the therapeutic alliance during tele-rehabilitation consultations. In addition, evidence suggests that therapeutic alliance is associated with better adherence to prescribed exercise. A cross-sectional study of 87 participants with musculoskeletal injuries found that the strongest predictor of adherence to home-based rehabilitation exercises was the therapeutic alliance between patients and the physical therapists treating them during in-person consultations [23].

### Rationale for This Study

Most existing studies evaluating relationships between therapeutic alliance and outcomes of physical therapy practice have focused on in-person consultations between patients and therapists. Thus, it is not clear if findings from such studies can be generalized to telephone-delivered models of physical therapy care, where patients and physical therapists have no physical or visual contact. Our research provides some evidence from qualitative studies that physical therapists and patients with OA perceive a strong alliance when consulting via video [24] and telephone [25,26]. In addition, we found that both patient and physical therapist ratings of the therapeutic alliance using a validated measure [27] were high, when consulting via telephone and generally in agreement with each other [28]. However, the relationship between therapeutic alliance and clinical outcomes from telephone-delivered physical therapy care remains unexplored. Thus, the aim of this study was to investigate whether the therapeutic alliance between patients and physical therapists is associated with self-reported clinical outcomes (including pain, function, fear of movement, quality of life, exercise adherence, treatment satisfaction, physical activity) at 6 and 12 months following telephone-delivered exercise and advice for people with knee OA.

## Methods

### Design

This exploratory study used data collected from physical therapists and patients in the intervention arm of a randomized controlled trial (RCT; Australian New Zealand Clinical Trials Registry (ANZCTRN) 12616000054415), which evaluated the effectiveness of incorporating physical therapist-delivered exercise advice and support into an existing musculoskeletal telephonic service delivered by nurses [10,29]. The funders played no role in the design, conduct, or reporting of this study. All participants provided written informed consent, and the institutional ethics committee approved the study.

### Patients

The intervention arm of the RCT included 87 randomized patients with knee OA. Inclusion criteria were meeting the National Institute for Health and Care Excellence OA clinical criteria (aged 45 years or over, with activity-related joint pain and morning stiffness ≤30 min) [5], an average knee pain of ≥4 on an 11-point numeric rating scale, and a history of knee pain for at least three months. The exclusion criteria have been published elsewhere [29]. Patients for the RCT were recruited from rural, regional, and metropolitan areas of Australia using advertisements on social media, on the radio, and in newspapers, through community organizations, and using previous volunteer databases.

### Physical Therapists

A total of 8 physical therapists were recruited in Victoria, Australia, to deliver the intervention for the trial. Selection criteria included a physical therapy qualification, at least two years of musculoskeletal professional experience, and current Australian registration to practice. Before the commencement of the trial, the physical therapists underwent a 2.5-day training program in the delivery of person-centered care and behavior change (delivered by HealthChange Australia) [30,31]. This involved the use of a set of practice principles to foster effective communication, techniques to identify and address barriers to behavior change, and a framework to guide decision making.

### Intervention

Details of the RCT have been published [29], including trial findings [10]. Patients in the intervention arm of the trial initially received a telephone call from a nurse as part of an existing musculoskeletal help line, where they received general information and advice about OA. Patients then received between 5 and 10 telephonic consultations from one of the eight physical therapists over a 6-month period (the same physical therapist provided all the consultations for each of their patients). During the initial consultation (approximately 40 min in length), the physical therapists helped increase patient knowledge and understanding of knee OA and the benefits of exercise. They worked with patients to devise goals and action plans that involved structured home-based strengthening exercise programs and/or physical activity plan. During follow-up consultations (approximately 20 min in length; the precise number of consultations was negotiated between the patients and their physical therapists), the physical therapists adjusted the program as necessary, while providing support using person-centered practice principles and behavior change techniques to help build patient confidence in their ability to undertake and adhere to an exercise program.

Patients were provided with a study folder containing information about OA and management, exercise instructions and access to a study website containing video demonstrations of each exercise. Patients were provided with three exercise resistance bands for home exercises.

### Outcome Measures

Outcome measures (collected at baseline, 6, and 12 months) in the RCT that were included in this secondary analysis were as follows:

1. Overall average knee pain in the past week (measured with a numeric rating scale ranging from 0 indicating no pain to 10, indicating the worst pain possible).
2. Physical function (measured using the Western Ontario and McMaster Universities Osteoarthritis Index [32] with scores ranging from 0 to 68, with lower scores indicating better function).
3. Self-efficacy (measured using the Arthritis Self-Efficacy Scale [33], total scores ranging from 3 to 30, with higher scores indicating greater self-efficacy).
4. Quality of life (using the assessment of quality of life [AQoL] instrument [34], with scores from -0.04 to 1.00, higher scores indicating better quality of life).
5. Global changes at 6 and 12 months (overall, pain, and function) via 7-point scales (terminal descriptors *much worse* to *much better*), as well as change in physical activity (descriptors *much less* to *much more*). Scores were dichotomized into 1 (improved or increased; those indicating *moderately better* or *more* or *much better* or *more*) and 0 (not improved or increased; those indicating *much worse*, *moderately worse*, *slightly worse*, or *no change*).
6. Satisfaction with care collected at 6 and 12 months via a 7-point scale (terminal descriptors *extremely unsatisfied* to *extremely satisfied*). Scores were dichotomized into *1* (satisfied; those indicating *moderately satisfied* or *extremely satisfied*) and *0* (not satisfied; those indicating *extremely unsatisfied*, *moderately unsatisfied*, *slightly unsatisfied*, or *neither satisfied* or *unsatisfied*).
7. Physical therapist-rated patient adherence to home exercise program collected at 6-months via an 11-point scale (terminal descriptors 0=*not at all* to 10=*completely as instructed*), only collected at 6 months.
8. Self-rated adherence to (a) prescribed exercises and (b) physical activity plan via an 11-point scale (terminal descriptors 0=*not at all* to 10=*completely as instructed*) rated at 6 and 12 months.

### Therapeutic Alliance Measures

Therapeutic alliance was measured using the Working Alliance Inventory-Short Form (WAI) [27,35], a commonly used valid and reliable measure of the alliance [27], which contains 12 statements relating to perceived trust and agreement between the therapist and the client (eg, “My patient/physical therapist and I agree about the things they/I will need to do in therapy to help improve my situation”). Statements were rated using a 7-point scale ranging from *never* feeling (or thinking) that way, to *always* feeling (or thinking) that way. The WAI has 3 subscales: (1) task (agreement on management methods being used; items 1, 2, 8, and 12); (2) bond (feelings of appreciation and trust; items 3, 5, 7, and 9), and (3) goal (agreement on aims and objectives of treatment; items 4, 6, 10, and 11), which are summed together to give a total score ranging from 12 to 84 (higher scores indicate a stronger alliance) [27,35].

As recommended [27], both patients and physical therapists completed the WAI separately, after their second consultation (approximately week 4 of the intervention). Although therapeutic alliance was also measured in the RCT at the end of the 6-month intervention, there was no significant change in total scores over time [28]. Thus, only scores obtained after the second consultation were used in this exploratory study.

### Data Analysis

Means and SDs of the patient and physical therapist characteristics and therapeutic alliance ratings were calculated. Separate regression models were used to investigate whether the therapeutic alliance was associated with each outcome. For each continuous outcome, linear regression models for the 6 and 12-month outcomes (change from baseline) were fit, with random effects for each patient to account for the two measurements. The baseline outcome measurement, where available, was included in the model, as were terms for patient (sex, age, self-efficacy at baseline, treatment expectations) and physical therapist characteristics (years of experience, previous experience delivering care remotely) that could potentially influence both the therapeutic alliance measure and outcomes at 6 and 12 months. The effect of therapeutic alliance on outcomes at 6 and 12 months was estimated by including terms for the outcome measurement time point, therapeutic alliance score, and an interaction between the two. Global change scores were dichotomized and analyzed using mixed-effects logistic regression models. Separate models were fit for the patient and physical therapist ratings of the alliance. As data for physical therapist ratings of adherence were only collected at 6 months, a standard linear regression model was fit. Analysis was performed using Stata (StataCorp, version 15.1).

## Results

### Characteristics of Patients With Knee OA

Most of the 87 patients (Table 1) were female (55/87, 63%) and lived in the metropolitan areas of Australia (48/87, 55%). The mean age of the patients was 62.4 years (SD 9.1), and at baseline, their mean knee pain was 6.0 (SD 1.5) on an 11-point numeric rating scale. Patients had a mean of 6.3 (SD 1.8) telephonic consultations during the trial and rated their therapeutic alliance a mean of 75.3 (SD 7.4) out of a maximum of 84.

**Table 1:** Characteristics of people with knee osteoarthritis (n=87).

Characteristic		Value
Female sex, n (%)		55 (63)
Age (years), mean (SD)		62.4 (9.1)
BMI (kg/m^2^), mean (SD)		31.1 (6.8)
**Location^a^, n (%)**		
	Metropolitan	48 (55)
	Nonmetropolitan	39 (45)
**Employment status, n (%)**		
	Working full- or part-time	37 (43)
	Unemployed or retired	50 (57)
**Education, n (%)**		
	Less than 3 years of high school	5 (6)
	3 years or more of high school	19 (23)
	Some tertiary training	21 (24)
	Graduated from university or polytechnic	24 (29)
	Any postgraduate study	15 (18)
Number of calls with physical therapist, mean (SD)		6.3 (1.8)
Therapeutic alliance (WAI^b^) at week 4, mean (SD)		75.3 (7.4)
Knee pain (NRS^c^) at baseline, mean (SD)		6.0 (1.5)
Physical function (WOMAC^d^) at baseline, mean (SD)		29.3 (10.1)
Self-efficacy (ASES^e^) at baseline, mean (SD)		20.2 (4.0)
Quality of life (AQoL^f^) at baseline, mean (SD)		0.7 (0.2)
Fear of movement (BFMS^g^) at baseline, mean (SD)		12.9 (3.5)
**Treatment expectations, n (%)**		
	No effect	0 (0)
	Minimal improvement	8 (9)
	Moderate improvement	46 (53)
	Large improvement	32 (37)
	Complete recovery	1 (1)

^a^Defined according to the Australian Statistical Geography Standard Remoteness Structure [36].

^b^WAI: Working Alliance Inventory; scores range from 12 to 84, where higher scores indicate a stronger alliance.

^c^NRS: numeric rating scale; ranges from 0 to 10, where lower scores indicate less pain.

^d^WOMAC: Western Ontario and McMaster Universities Osteoarthritis Index; ranges from 0 to 68, where lower scores indicate better function.

^e^ASES: Arthritis Self-Efficacy Scale: scores range from 3 to 30, where higher scores indicate greater self-efficacy.

^f^AQoL: Assessment of quality of life instrument, ranges from −0.04 to 1.0, where higher scores indicate better quality of life.

^g^BFMS: Brief Fear of Movement Scale; ranges from 0 to 24, where higher scores indicate lower fear of movement.

### Characteristics of Physical Therapists

Half of the 8 physical therapists (Table 2) were male and 63% (5/8) worked exclusively in private physical therapy settings. Collectively, physical therapists had a mean of 13.8 (SD 8.2) years of clinical experience, and none had experience delivering care via telephone, although 25% (2/8) had experience doing so via Skype. Physical therapists consulted with a mean of 10.5 (SD 2.1) trial patients each, and rated the therapeutic alliance as a mean of 71.0 (SD 5.5) out of a maximum of 84.

**Table 2:** Characteristics of physical therapists (n=8).

Characteristic		Value
Female, n (%)		50 (50)
Age (years), mean (SD)		35.4 (8.2)
Clinical experience (years), mean (SD)		13.8 (8.2)
Number of patients consulted with in the trial, mean (SD)		10.5 (2.1)
**Work setting, n (%)**		
	Both private and public	2 (25)
	Private	5 (63)
	Public	1 (12)
**Previous experience delivering care remotely, n (%)**		
	None	6 (75)
	Yes (via Skype)	2 (25)
	Yes (via telephone)	0 (0)
**Postgraduate training in knee osteoarthritis, n (%)**		
	Yes	3 (37)
	No	5 (63)
**Postgraduate training in exercise, n (%)**		
	Yes	7 (88)
	No	1 (12)
**Postgraduate training in behavior change^a^, n (%)**		
	Yes	3 (37)
	No	5 (63)
Therapeutic alliance (WAI^b^) at week 4, mean (SD)		71.0 (5.5)

^a^Excluding trial-specific training in person-centered principles and behavior change techniques.

^b^WAI: Working Alliance Inventory; scores range from 12 to 84, where higher scores indicate a stronger alliance.

### Association of Patient-Rated Therapeutic Alliance With Outcomes

Associations between patient ratings of therapeutic alliance and continuous and binary outcomes at 6 and 12 months are displayed in Tables 3 and 4, respectively. Data suggest that patient-rated therapeutic alliance was associated with some outcomes at 6 months. Regression coefficients show that a one-unit increase in the therapeutic alliance score was associated with (1) a −0.10 (95% CI −0.16 to −0.03) unit improvement in overall average knee pain measured via a numeric rating scale; (2) a −0.13 (95% CI −0.23 to −0.04) unit worsening in fear of movement; (3) a 0.16 (95% CI 0.04 to 0.28) unit improvement in self-efficacy; (4) increased odds of global improvement in physical function (odds ratio [OR] 1.21, 95% CI 1.04-1.39), and (5) increased odds of a global improvement overall (OR 1.26, 95% CI 1.06 to 1.51).

Data suggest that patient-rated therapeutic alliance was associated with some outcomes at 12 months. A one-unit increase in the therapeutic alliance score was associated with (1) a 0.15 (95% CI 0.03 to 0.27) unit improvement in self-efficacy; (2) increased odds of a global improvement in pain (OR 1.14, 95% CI 1.01 to 1.30); (3) increased odds of a global improvement in physical function (OR 1.19, 95% CI .03 to 1.37), and (4) increased odds of a global improvement overall (OR 1.21, 95% CI 1.02 to 1.42).

**Table 3:** Associations between patient and physical therapist ratings of the therapeutic alliance and changes in continuous outcomes at 6 and 12 months.^a^

Outcome		6 months		12 months	
		Regression coefficient^b^ (95% CI)	*P* value	Regression coefficient^b^ (95% CI)	*P* value
**Patient rating of therapeutic alliance**					
	Overall average knee pain (NRS^c^)	−0.10 (−0.16 to −0.03)	<.01	−0.06 (−0.13 to 0.00)	.06
	Physical function (WOMAC^d^ C)	−0.10 (−0.40 to 0.20)	.52	−0.13 (−0.43 to 0.18)	.42
	Fear of movement (BFMS^e^)	−0.13 (−0.23 to −0.04)	<.01	−0.08 (−0.18 to 0.01)	.08
	Health-related quality of life (AQoL^f^)	0.01 (0.01 to −0.01)	.43	0.01 (−0.01 to 0.01)	.42
	Self-efficacy (total; ASES^g^)	0.16 (0.04 to 0.28)	.01	0.15 (0.03 to 0.27)	.02
	Overall self-rated adherence to prescribed exercise	0.09 (−0.02 to 0.20)	.11	0.04 (−0.07 to 0.15)	.46
	Self-rated adherence to prescribed physical activity	0.08 (−0.02 to 0.18)	.10	0.07 (−0.03 to 0.17)	.15
	Physical therapist-rated patient adherence	0.02 (−0.04 to 0.09)	.49	—^h^	—
**Physical therapist rating of therapeutic alliance**					
	Overall average knee pain (NRS)	−0.02 (−0.11 to 0.06)	.55	−0.06 (−0.14 to 0.02)	.15
	Physical function (WOMAC C)	−0.14 (−0.50 to 0.23)	.47	−0.03 (−0.40 to 0.35)	.89
	Fear of movement (BFMS)	−0.07 (−0.20 to 0.06)	.28	−0.08 (−0.21 to 0.04)	.20
	Health-related quality of life (AQoL)	0.01 (−0.01 to 0.01)	.25	0.01 (0.0003 to 0.01)	.04
	Self-efficacy (total; ASES)	0.06 (−0.11 to 0.23)	.50	0.04 (−0.13 to 0.22)	.65
	Overall self-rated adherence to prescribed exercise	0.03 (−0.11 to 0.18)	.65	−0.03 (−0.17 to 0.11)	.67
	Self-rated adherence to prescribed physical activity	0.03 (−0.10 to 0.16)	.67	−0.01 (−0.14 to 0.12)	.90
	Physical therapist-rated patient adherence	0.05 (−0.03 to 0.13)	.24	—	—

^a^Calculated as follow-up (6 or 12 months) minus baseline.

^b^Regression coefficients are not standardized. Regression models were adjusted and baseline outcome measures, patient variables (gender, age, self-efficacy at baseline, treatment expectations), and physical therapist variables (years of experience, previous experience delivering care remotely) were included as covariates.

^c^NRS: numeric rating scale; ranges from 0 to 10. Negative coefficients indicate that a stronger therapeutic alliance is associated with reduced pain over time.

^d^WOMAC: Western Ontario and McMaster Universities Osteoarthritis Index; ranges from 0 to 68. Negative coefficients indicate that a stronger therapeutic alliance is associated with reduced functional impairment over time.

^e^BFMS: Brief Fear of Movement Scale; ranges from 0 to 24. Positive coefficients indicate that a stronger therapeutic alliance is associated with an improvement in fear of movement over time.

^f^AQoL: Assessment of quality of life instrument, ranges from −0.04 to 1.0. Positive coefficients indicate that a stronger therapeutic alliance is associated with improvement in quality of life over time.

^g^ASES: Arthritis Self-Efficacy Scale: scores range from 3 to 30. Positive coefficients indicate that a stronger therapeutic alliance is associated with improvement in self-efficacy over time.

^h^—: Outcome measure not collected at 12 months.

**Table 4:** Associations between patient and physical therapist ratings of the therapeutic alliance and binary outcomes at 6 and 12 months.^a^

Outcome		6 months		12 months	
		OR^b^ (95% CI)	*P* value	OR^b^ (95% CI)	*P* value
**Patient rating of therapeutic alliance**					
	Improved pain	1.12 (0.99 to 1.26)	.08	1.14 (1.01 to 1.30)	.03
	Improved physical function	1.21 (1.04 to 1.39)	.01	1.19 (1.03 to 1.37)	.02
	Improved overall	1.26 (1.06 to 1.51)	.01	1.21 (1.02 to 1.42)	.03
	Satisfied with care received	1.12 (0.95 to 1.33)	.17	1.10 (0.96 to 1.25)	.18
	Increased physical activity levels	1.09 (0.96 to 1.24)	.18	1.11 (0.97 to 1.26)	.12
**Physical therapist rating of therapeutic alliance**					
	Improved pain	1.07 (0.93 to 1.24)	.33	1.10 (0.95 to 1.28)	.21
	Improved physical function	1.13 (0.95 to 1.33)	.17	1.04 (0.89 to 1.22)	.63
	Improved overall	1.13 (0.94 to 1.36)	.18	1.09 (0.92 to 1.31)	.32
	Satisfied with care received	1.16 (0.94 to 1.43)	.16	0.89 (0.73 to 1.09)	.26
	Increased physical activity levels	1.04 (0.90 to 1.20)	.57	1.06 (0.92 to 1.23)	.39

^a^Regression models were adjusted and baseline outcome measures, patient variables (gender, age, self-efficacy at baseline, and treatment expectations), and physical therapist variables (years of experience and previous experience delivering care remotely) were included as covariates.

^b^OR: odds ratio; ORs >1 indicate greater odds of reporting improvement in outcome or satisfaction with care with a stronger therapeutic alliance.

### Association of Physical Therapist Ratings of the Therapeutic Alliance With Outcomes

Associations between physical therapist ratings of the therapeutic alliance and continuous and binary outcomes at 6 and 12 months are displayed in Tables 3 and 4, respectively. There was no evidence of an association between the physical therapist–rated therapeutic alliance and any outcomes at 6 months; only one outcome at 12 months was associated. A one-unit increase in therapeutic alliance was associated with a regression coefficient of 0.01 (95% CI 0.0003 to 0.01) unit improvement in health-related quality of life.

## Discussion

### Principal Findings

The aim of this study was to investigate whether the therapeutic alliance between patients and physical therapists during telephonic consultations was associated with outcomes following exercise and advice for people with knee OA. The findings suggest that patient-rated therapeutic alliance was weakly associated with some outcomes at 6 and 12 months, including improvements in pain, self-efficacy, global function, and overall global improvement, in addition to a worsening in fear of movement. The data indicated that associations between physical therapist-rated therapeutic alliance and outcomes were not meaningful. The observed relationships were generally weak and thus unlikely to be clinically significant.

### Comparison With Earlier Work

This is the first study to investigate the relationship between therapeutic alliance and clinical outcomes following telephone-delivered physical therapy care in adults with OA. Existing reviews focusing on traditional, in-person consultations among those with musculoskeletal conditions have found that a stronger therapeutic alliance between the patient and their physical therapist is associated with improved outcomes, including better adherence to physical-therapist–prescribed exercise and physical activity [37], improved global effects (pain, physical function, disability) [21], and greater treatment satisfaction [37,38]. We also found some evidence of an association with improved global effects; however, our data did not indicate a strong association between therapeutic alliance and exercise adherence or treatment satisfaction. The reason remains unclear. However, we measured adherence and satisfaction using self-reported questionnaires and analyzed associations with a valid and reliable measure of therapeutic alliance. Other studies included in a review by Babatunde et al, [37] have qualitatively explored the relationship between therapeutic alliance and adherence, or used unvalidated custom-developed measures of alliance, which makes comparisons with our findings difficult. In addition, we found that a higher therapeutic alliance was associated with greater improvements in self-efficacy over time. To our knowledge, no previous studies have examined the association between therapeutic alliance and changes in self-efficacy. Intuitively, this finding makes sense, in that greater perceived agreement on tasks and goals and a greater perceived bond with therapists is related to improvements in confidence and belief in one’s ability. Unexpectedly, we found that a higher patient-perceived therapeutic alliance was associated with worsening of fear of movement at 6 months, but at 12 months, the direction of the association was uncertain. In the overarching clinical trial, fear of movement worsened over time in both the intervention and control groups, with no differences in change between groups [10]. To our knowledge, no other studies have examined the association between therapeutic alliance and change in fear of movement after treatment; thus, further research is required to confirm this finding.

Our findings are broadly similar to those of previous research exploring therapeutic alliance in tele-rehabilitation consultations with clinicians outside of the physical therapy profession. One study of 22 adolescents (mean age 15 years) with idiopathic arthritis who received care via 12 telephonic consultations from trained nonprofessional health coaches over 12 weeks found that therapeutic alliance was correlated with improved treatment outcomes, including decreased pain [39]. However, the authors only reported correlation coefficients, which makes comparisons with the magnitude of association observed in our study difficult. Other populations of people with psychological disorders (eg, post-traumatic stress disorder, anxiety, depression, cancer stress) have found that therapeutic alliances during therapist-led remotely delivered (ie, via video or telephone) cognitive behavioral therapy is associated with improvements in outcomes (eg, reduced symptoms of depression and anxiety, increased compliance) at 5 to 18 weeks [40-42]. However, given paucity of evidence, particularly in remotely delivered physical therapy, further research is required.

Although we observed associations between therapeutic alliance and outcomes, the coefficients were very small and confidence intervals contained values that were close to zero. Thus, the clinical significance of our observed relationships is unclear. A single unit increase in therapeutic alliance score (measured on a scale of 12 to 84, with an SD of 7.4) corresponded to a very small, 0.10-unit improvement in overall average knee pain (measured on an 11-point numeric rating scale) at 6 months. This magnitude of change is similar to that observed by Ferreira et al [43], who investigated associations between therapeutic alliance and clinical outcomes following 12 in-person consultations with physical therapists over 8 weeks for patients with low back pain. This suggests that consulting via telephone does not change the relationship between therapeutic alliance and outcomes when compared with being in-person. Ferreira et al [43] found that a 1-SD increase in therapeutic alliance score (measured using a different version of the WAI to the one used in our study) corresponded to a 0.6-unit improvement in pain (measured on an 11-point numeric rating scale). For context, the minimal clinically important difference for pain following interventions for people with OA is an absolute change of 2.0 units on a numeric rating scale [44], which suggests that therapeutic alliance may not have a clinically significant impact on pain. In quality of life, a 1-SD increase in physical therapist-rated therapeutic alliance score in our study corresponds to a 0.055-unit improvement in quality of life, approximating the estimated minimal clinically important difference of 0.06 on the AQoL [45]. Minimal clinically important differences for other outcome measures that we found were associated with therapeutic alliance (including self-efficacy and fear of movement) are unknown [46], and as such, the clinical significance of associations with these outcomes is unclear.

Our patient and physical therapist ratings of the therapeutic alliance were high [28], and the small SD suggests that there was no significant variability in scores. This does not appear to be unique to our sample, as other studies investigating therapeutic alliances in physical therapy or tele-rehabilitation have also observed high scores with low variability in their sample [39,43]. A variety of tools have been used to evaluate therapeutic alliance [37]; however, none have been validated for use in musculoskeletal physical therapy settings. These existing tools may not necessarily capture domains of care that are important in physical therapy contexts [47] and have been found to demonstrate a ceiling effect [48]. Therefore, the development of measures that are validated in musculoskeletal physical therapy settings is important.

Our study is relevant to clinicians and researchers. The findings suggest that the strength of the therapeutic alliance with the physical therapist as perceived by the patient is associated with some clinical outcomes after telephonic consultations focused on exercise management. Thus, physical therapists should be mindful about the therapeutic alliance they build with their patients. To enhance the therapeutic alliance, it has been recommended that clinicians focus on fostering person-centered interactions with patients, including offering emotional support and facilitating patient involvement in decision-making [49-51]. It is also important to acknowledge, however, that we currently do not understand the clinical importance of the observed associations between therapeutic alliance and outcomes, and it is also not clear which strategies are best to increase therapeutic alliance. Further research is required to determine what specific components of care or clinician skills may need to be modified to enhance therapeutic alliance, and whether it is practical for physical therapists or other clinicians to adapt such skills in clinical practice. In addition, further research is required to investigate whether clinician experience with or training in remotely delivered care influences therapeutic alliance during telephonic consultations. Studies that include manipulation of therapeutic alliance may provide more insight into its importance in clinical practice. For example, Fuentes et al [52] randomized 117 people with chronic low back pain to enhanced and limit therapeutic alliance groups, where physical therapists either did not engage in conversation with patients and left the room during interferential current therapy (limited alliance group) or engaged in active listening and used empathetic language and encouragement (enhanced alliance group). They found that those allocated to the enhanced therapeutic alliance groups reported significantly greater improvements in pressure pain threshold and pain than those in the limited alliance group immediately after the treatment session.

Future research should consider evaluating relationships between therapeutic alliance and clinical outcomes in real-world clinical practice, as both alliance and clinical outcomes may be more varied than observed within the context of a clinical trial. Importantly, we found that physical therapist ratings of therapeutic alliance were generally not related to clinical outcomes, suggesting that their own perceptions of the alliance may not be as important as those of the patient. Our study was the first to investigate the relationship between therapeutic alliance and clinical outcomes following telephone-delivered physical therapy care in adults with OA, and thus further research is required to compare therapeutic alliance during tele-rehabilitation and traditional in-person consultations, and how it moderates treatment outcomes.

### Limitations

Our study has some limitations. As with any study, there is a risk of type 1 error. However, in accordance with recommendations from the American Statistical Association [53], we did not interpret our results on the basis of statistical significance alone, instead considering the clinical significance of the findings. Before commencement of the trial, all trial physical therapists underwent training in person-centered care and behavior change techniques [30]. Our findings may not be generalizable to other physical therapists in the community who have not undergone such training. Most (5/8, 63%) of our physical therapists worked in private health care settings, where patients typically incur out-of-pocket costs for services. This broadly reflects the physical therapy workforce in Australia, where more than 60% of therapists work in private settings [54]. Thus, our findings may not be generalizable to other countries where physical therapists may work in alternate health care settings. We used the WAI to measure the therapeutic alliance between patients and physical therapists; however, this tool has not been validated for use in musculoskeletal physical therapy practice, and similar measures of therapeutic alliance have been found to demonstrate a ceiling effect [48]. Finally, a limitation of our study is that our dependent variables (clinical outcomes) were measured via participant-reported outcome measures. It is unclear if our findings may have differed had we used objectively measured outcomes (such as performance tests of physical function) which is an area where future research may be warranted.

### Conclusions

In conclusion, higher patient ratings but not higher physical therapist ratings of the therapeutic alliance were weakly associated with improvements in some clinical outcomes. Although these findings suggest that patients who perceive a stronger alliance with their physical therapist may achieve some better clinical outcomes, the observed relationships were generally weak and unlikely to be clinically significant. Limitations include the fact that measures of therapeutic alliance have not been validated for use in musculoskeletal physical therapy settings. There was a risk of type 1 error; however, findings were interpreted based on clinical significance rather than statistical significance alone.

## Abbreviations

AQoL: assessment of quality of life
NHMRC: National Health and Medical Research Council
OA: osteoarthritis
OR: odds ratio
RCT: randomized controlled trial
WAI: Working Alliance Inventory
